# Akt is required for Stat5 activation and mammary differentiation

**DOI:** 10.1186/bcr2640

**Published:** 2010-09-17

**Authors:** Chien-Chung Chen, Robert B Boxer, Douglas B Stairs, Carla P Portocarrero, Rachel H Horton, James V Alvarez, Morris J Birnbaum, Lewis A Chodosh

**Affiliations:** 1 Department of Cancer Biology, University of Pennsylvania School of Medicine, 421 Curie Boulevard, Philadelphia, PA 19104, USA; 2 Abramson Family Cancer Research Institute, University of Pennsylvania School of Medicine, 421 Curie Boulevard, Philadelphia, PA 19104, USA; 3 Department of Medicine, University of Pennsylvania School of Medicine, 3620 Hamilton Walk, Philadelphia, PA 19104, USA; 4 Department of Cell and Developmental Biology, University of Pennsylvania School of Medicine, 421 Curie Boulevard, Philadelphia, PA 19104, USA

## Abstract

**Introduction:**

The Akt pathway plays a central role in regulating cell survival, proliferation and metabolism, and is one of the most commonly activated pathways in human cancer. A role for Akt in epithelial differentiation, however, has not been established. We previously reported that mice lacking *Akt1*, but not *Akt2*, exhibit a pronounced metabolic defect during late pregnancy and lactation that results from a failure to upregulate Glut1 as well as several lipid synthetic enzymes. Despite this metabolic defect, however, both *Akt1*-deficient and *Akt2*-deficient mice exhibit normal mammary epithelial differentiation and Stat5 activation.

**Methods:**

In light of the overlapping functions of Akt family members, we considered the possibility that Akt may play an essential role in regulating mammary epithelial development that is not evident in *Akt1*-deficient mice due to compensation by other Akt isoforms. To address this possibility, we interbred mice bearing targeted deletions in *Akt1 *and *Akt2 *and determined the effect on mammary differentiation during pregnancy and lactation.

**Results:**

Deletion of one allele of *Akt2 *in *Akt1*-deficient mice resulted in a severe defect in Stat5 activation during late pregnancy that was accompanied by a global failure of terminal mammary epithelial cell differentiation, as manifested by the near-complete loss in production of the three principal components of milk: lactose, lipid, and milk proteins. This defect was due, in part, to a failure of pregnant *Akt1^-/-^*;*Akt2^+/- ^*mice to upregulate the positive regulator of Prlr-Jak-Stat5 signaling, Id2, or to downregulate the negative regulators of Prlr-Jak-Stat5 signaling, caveolin-1 and Socs2.

**Conclusions:**

Our findings demonstrate an unexpected requirement for Akt in Prlr-Jak-Stat5 signaling and establish Akt as an essential central regulator of mammary epithelial differentiation and lactation.

## Introduction

The serine/threonine kinase Akt is a critical downstream effector in multiple signal transduction pathways and regulates cellular proliferation, survival, and metabolism. Consistent with this, Akt is inappropriately activated in a wide range of human cancers [1,2]. Three Akt isoforms (Akt1, Akt2, and Akt3) are present in mammals, and targeted deletion of each gene has revealed distinct as well as overlapping functions in cellular physiology. *Akt1*^-/- ^mice exhibit increased perinatal mortality and a modest growth defect. *Akt2*^-/- ^mice are viable but develop insulin resistance and a diabetes-like phenotype, whereas *Akt3*^-/- ^mice have normal glucose homeostasis but decreased brain size [3-7].

Given the high degree of homology among Akt isoforms, the possibility that these three proteins play redundant roles has been addressed by generating mice deficient for multiple isoforms. This has revealed that *Akt1*^-/-^;*Akt2*^-/- ^mice display perinatal lethality, reduced growth, and defects in skin and bone development, as well as adipogenesis [8]. *Akt1*^-/-^;*Akt3*^-/- ^mice die during embryonic development at embryonic day 12, and *Akt1*^-/-^;*Akt3*^+/- ^mice exhibit developmental abnormalities in multiple organs that result in the death of 90% of mice shortly after birth [9]. In contrast, *Akt2*^-/-^;*Akt3*^-/- ^mice are born in Mendelian ratios, but are significantly smaller than wild-type littermates [10]. These results indicate that individual Akt isoforms play both unique and overlapping roles in development and physiology, and further suggest that a critical threshold of Akt activity may be required to produce a given cellular output.

Similar to Akt, the Stat5 pathway plays a central role in regulating cellular function and is activated in response to a wide range of stimuli, including growth factors, cytokines, and hormones. Stat5 regulates cell proliferation, survival, and differentiation through direct activation of target genes. Stat5 is encoded by two closely related genes, Stat5a and Stat5b. While constitutive deletion of both genes leads to embryonic lethality [11], conditional knockouts have revealed tissue-specific functions of Stat5 [11-14].

The best-characterized role of Stat5 in normal physiology is the regulation of pregnancy-induced mammary epithelial development, where it is essential for the proliferation, differentiation, and survival of mammary epithelial cells [11]. Analysis of a variety of Stat5 mutant alleles in mice has revealed that Stat5 deficiency in the mammary gland leads to a near-complete loss of lobuloalveolar development, a reduction in expression of milk protein genes during pregnancy, and lactation failure [11,15-18]. Stat5 deletion during pregnancy results in the death of differentiated mammary epithelial cells, with further experiments suggesting that the requirement for Stat5 is required for the survival of alveolar luminal progenitor cells during pregnancy-induced lobuloalveolar development [11,19].

Constitutive activation of Akt in the mammary epithelium promotes the precocious accumulation of intracellular lipid droplets during pregnancy and delays post-weaning involution by inhibiting apoptosis [20-22]. Consistent with its upstream role as a negative regulator of Akt activity, *Pten*-deficient mice exhibit delayed mammary involution and reduced apoptosis [23], whereas forced expression of Pten in the mammary gland results in impaired lactation due to decreased mammary epithelial proliferation and increased apoptosis during pregnancy [24].

We recently investigated the role of Akt in mammary development by examining mice bearing targeted deletions in either *Akt1 *or *Akt2 *[25]. We found that loss of both alleles of *Akt1 *results in failure of the coordinated metabolic response required for the establishment of lactation at parturition, including increased glucose uptake and lipid synthesis, which in turn results in decreased milk production. In contrast, deletion of both alleles of *Akt2 *had no discernible effect on lactation. Notably, despite the requirement for Akt1 in the metabolic control of the lactating mammary gland, mammary epithelial differentiation, proliferation, and survival were unaffected during pregnancy in *Akt1*^-/- ^mice.

In light of the overlapping functions of Akt isoforms, we considered the possibility that Akt may play an essential role in regulating mammary epithelial development that is not evident in *Akt1*^-/- ^mice due to compensation by other Akt isoforms. To address this issue, we interbred mice bearing targeted deletions in *Akt1 *and *Akt2 *in order to determine the effect on mammary differentiation. We find that deletion of one allele of *Akt2 *in *Akt1*^-/- ^mice results in a severe defect in terminal mammary epithelial differentiation and lactation failure due to a loss of prolactin-mediated Stat5 activation. Notably, this defect occurred in the absence of changes in pregnancy-induced lobuloalveolar development, including proliferation, apoptosis or acinar formation. As such, the defects observed in *Akt1*^-/-^;*Akt2*^+/- ^mice reflect the abrogation of Stat5 signaling and the molecular program of terminal differentiation in the mammary gland, a program that is intact in *Akt1*-deficient mice. Our observations demonstrate an unexpected requirement for Akt in Prlr-Jak-Stat5 signaling in the mammary gland, and establish Akt as an essential regulator of differentiation, metabolism and lactation in the mammary gland.

## Materials and methods

### Animals

*Akt1*^-/- ^and *Akt2*^-/- ^mice of C57BL/6 genetic background were generated and provided by Dr Morris Birnbaum (Howard Hughes Medical Institute, University of Pennsylvania, Philadelphia, PA, USA) [5,6]. Mice were housed and maintained according to Institutional Animal Use and Care Committee guidelines. For timed pregnancies, the morning of the observed virginal plug was counted as day 0.5. At given time points, animals were killed by carbon dioxide asphyxiation and the mammary gland tissues were harvested and snap-frozen on dry ice, fixed in 4% paraformaldehyde in 1 × PBS (4% paraformaldehyde (PFA)) or frozen in Optimal Cutting Temperature (OCT) compound for further analysis.

The determination of pup weight, milk volume and pup mortality among various knockout mice has been described previously [25,26]. All experiments and experimental methods related to the use of animals were approved by the University of Pennsylvania Institutional Animal Use and Care Committee.

### Antibodies

The following rabbit polyclonal antibodies were used in the study: phospho-S6 (Ser235/236), S6, and Akt (Cell Signaling Technology, Danvers, MA, USA), suppressor of cytokine signaling 2(Socs2) (Zymed Laboratories, South San Francisco, CA, USA), inhibitor of DNA binding 2 (Id2) (Santa Cruz Biotechnology, Santa Cruz, CA, USA), and mouse milk-specific proteins (Nordic Immunological Laboratories, Tilburg, Netherlands). The following mouse monoclonal antibodies were used: phospho-Stat5a/b (Tyr694/Tyr699) and Stat5a/b (Upstate, Lake Placid, NY, USA), β-tubulin (BioGenex, San Ramon, CA, USA), caveolin-1 (BD Biosciences, San Diego, CA, USA) and Gata-3 (Santa Cruz Biotechnology). The goat anti-Elf5 polyclonal antibody was purchased from Santa Cruz Biotechnology.

The rabbit anti-Npt2b polyclonal antibody was generously provided by Dr Jim Turner (National Institute of Dental and Craniofacial Research, National Institute of Health, Bethesda, MD, USA). The rat anti-cytokeratin 8 (CK8) was purchased from the Developmental Studies Hybridoma Bank (University of Iowa, Iowa City, IA, USA).

### Mammary gland whole-mount and histological analysis

The abdominal mammary glands fixed in 4% PFA were either stained in carmine/aluminum potassium sulfate for whole mounts as previously described [27] or embedded in paraffin wax. For histological analysis, 5 μm tissue sections were cut, dewaxed in xylene, rehydrated, and stained with H & E.

### Immunofluorescence analysis

After paraffin-embedded 5 μm tissue sections were cleared and rehydrated, sections were subjected to antigen retrieval performed by heating treatment in an antigen unmasking solution (Vector Laboratories, Burlingame, CA, USA). Subsequently, sections were incubated in blocking solution consisting of 5% BSA and 10% (v/v) normal goat serum in PBS at room temperature for 1 hour. The primary antibodies phospho-Stat5 (1:100), Npt2b (1:300) and anti-CK8 (1:100) were then applied and incubated at 4°C overnight. Appropriate fluorescein-conjugated secondary antibodies (Molecular Probes, Eugene, OR, USA) were applied for 1 hour, counterstained with 5 mg/ml Hoechst 33258 (1:10,000), and mounted with Immu-mount (Thermo Scientific, Pittsburgh, PA, USA).

Stained sections were examined using a Leica microscope (Model DM5000B; Leica Microsystems, Bannockburn, IL, USA) equipped with a mercury lamp and FITC (L5), Texas Red (TX2) and Hoechst/DAPI (A4) filter cubes. Images were acquired by a digital camera (Leica DFC350FX) operated and analyzed with Image-Pro Express software and processed with the Photoshop program.

### Northern blot, *in situ *hybridization and quantitative RT-PCR analysis

Total RNA isolation from snap-frozen abdominal and inguinal mammary tissues without lymph nodes, preparation of radioactively labeled cDNA probes, northern blots and *in situ *hybridization were performed as previously described [28]. The cDNA probes for northern hybridization correspond to *β-casein *(nucleotides 181 to 719), whey acidic protein (*WAP*) (nucleotides 131 to 483), *ε-casein *(nucleotides 83 to 637), *α-lactalbumin *(nucleotides 174 to 700) and *CK18 *(nucleotides 589 to 1287). The sequence of an oligomer used to detect 18S rRNA is CGGAACTACGACGGTATCTG.

Single-stranded cDNA for quantitative RT-PCR analysis was generated by the high-capacity cDNA reverse transcription kit (Applied Biosystems, Carlsbad, CA, USA). Quantitative RT-PCR was performed using the TaqMan-based ABI 7900HT fast real-time PCR system according to the manufacturer's instructions (Applied Biosystems). The probes used were *Aldoc *Mm01298111_g1, *Fads1 *Mm00507605_m1, *Elovl5 *Mm00506717_m1, *Prlr *Mm00599957_m1, and *cytokeratin 18 *Mm01601706_g1.

### Western blot analysis

Frozen abdominal and inguinal mammary tissues (lymph nodes removed) were homogenized in lysis buffer (1% Triton X-100, 50 mM Tris-HCl, pH 7.4, 150 mM NaCl, 1 mM ethylenediamine tetraacetic acid (EDTA), 50 mM sodium fluoride (NaF), 3 mM sodium pyrophosphate, and 5 mM β-glycerolphosphate) supplemented with protease inhibitor cocktail (Roche, Indianapolis, IN, USA) and were subjected to western blot analysis, performed as described previously [25]. The following primary antibodies were used: phospho-S6 (Ser235/236) (1:1,000), S6 (1:1,000), Akt (1:1,000), phospho-Stat5a/b (Tyr694/Tyr699) (1:500), Stat5a/b (1:500), mouse milk-specific proteins (1:20,000), β-tubulin (1:1,000), caveolin-1 (1:1,000), Socs2 (1:150), Id2 (1:100), and Elf5 (1:100). Chemiluminescence was detected with horseradish peroxidase-conjugated goat anti-rabbit or mouse secondary antibodies at a dilution of 1:5,000, and was developed in the ECL plus system based on the manufacturer's protocol (Amersham Biosciences, Piscataway, NJ, USA) followed by exposure to X-ray film (Kodak, Rochester, NY, USA). All experiments were independently repeated three times. Only the representative images are shown. Densitometry for western blots was carried out with the Photoshop program.

### Lactose analysis

Lactose in the mammary gland was measured by subjecting 20 μg tissue lysates to the lactose assay kit as per the manufacturer's instructions (MBL, Woburn, MA, USA). The lactose level was calculated by subtracting the free galactose level from the total galactose level.

### Mammary gland culture

The whole-organ culture of the mammary gland was as described previously but with slight modifications [26]. Briefly, the lymph node-free abdominal and inguinal glands of *Akt1*^+/+^;*Akt2*^+/+ ^mice and *Akt1*^-/-^;*Akt2*^+/- ^mice at day 18.5 of pregnancy were aseptically collected and minced into fine pieces (~2 mm). Tissues were incubated in Waymouth's serum-free medium (Invitrogen, Carlsbad, CA, USA) supplemented with 20 mM HEPES, 4 mM glutamine, 5 μg/ml insulin, and 1 μg/ml hydrocortisone. The tissues were replaced with fresh culture medium daily to remove hormones carried from mice, and the mammary tissues were cultured for 5 days followed by growth-factor starvation overnight. The tissues treated with 0.2 μg/ml prolactin for the indicated periods were collected.

### Statistical analysis

Data are presented as the mean ± standard error of the mean (SEM). Statistical analysis was calculated by Student's *t *test unless otherwise indicated.

## Results

### Akt is required for lactation

Homozygous deletion of either *Akt1 *or *Akt2 *has no effect on mammary epithelial cell differentiation [25]. *Akt1 *is required in an isoform-specific manner, however, for coordinating multiple metabolic pathways in the mammary gland during the transition from pregnancy to lactation [25]. The inability of lactating *Akt1*^-/- ^mice to secrete normal amounts of milk arises from a failure to upregulate Glut1, glucose uptake, and lipid synthesis, as well as a failure to downregulate lipid catabolism [25]. In contrast, Akt2 is entirely dispensable for the metabolic response of the mammary gland to lactation.

Since *Akt1 *and *Akt2 *are expressed in the same cell types in the mammary gland [25], we considered the possibility that they might have compensatory roles in development. As deletion of one *Akt3 *allele in *Akt1*-deficient mice results in perinatal lethality, we generated mice with combined loss of *Akt1 *and *Akt2 *alleles. Because *Akt1*^-/-^;*Akt2*^-/- ^mice die shortly after birth, we compared the ability of *Akt1*^-/-^;*Akt2*^+/- ^mice and *Akt1*^-/-^;*Akt2*^+/+ ^mice to support the growth and survival of nursing pups.

**Figure 1:**
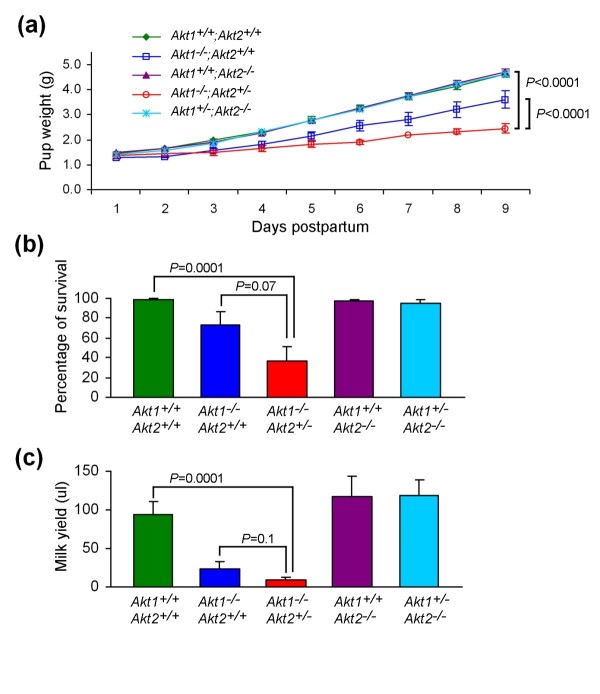
**Deletion of one *Akt2 *allele in *Akt1*-deficient mice exacerbates their lactation defect**. **(a) **Postpartum weight gain in pups nursed by Akt foster mice. Average daily pup weights for litters nursed by *Akt1*^+/+^;*Akt2*^+/+ ^mice, *Akt1*^-/-^;*Akt2*^+/+ ^mice, *Akt1*^+/+^;*Akt2*^-/- ^mice, *Akt1*^-/-^;*Akt2*^+/- ^mice, or *Akt1*^+/-^;*Akt2*^-/- ^mice. Statistical analysis was calculated by mixed model with first-order autoregressive covariance structure. **(b) **Pup survival in litters nursed by Akt foster mice. Pup survival was defined as the percentage of mice that survived to postpartum day 9. **(c) **Milk yield collected from the thoracic and abdominal mammary glands of mice with indicated Akt genotypes at day 9 of lactation after oxytocin stimulation.

In agreement with our previous report [25], pups nursed by *Akt1*^-/-^;*Akt2*^+/+ ^mothers, but not those nursed by *Akt1*^+/+^;*Akt2*^-/- ^mothers, exhibited growth retardation compared with pups nursed by wild-type mice (Figure 1a). Deletion of one allele of *Akt2 *on an *Akt1*-deficient background exaggerated the growth defect observed in pups nursed by *Akt1*-deficient mice (*P *< 0.0001). In contrast, deletion of one *Akt1 *allele on an *Akt2*-deficient background had no effect on the ability of lactating mothers to support pups (Figure 1a). By postpartum day 4, pups nursed by *Akt1*^-/-^;*Akt2*^+/- ^mice weighed significantly less than those nursed by *Akt1*^-/-^;*Akt2*^+/+ ^mice. By postpartum day 9, the average weight of pups nursed by *Akt1*^-/-^;*Akt2*^+/- ^mothers was two-thirds that of pups nursed by *Akt1*^-/-^;*Akt2*^+/+ ^mothers and one-half that of pups nursed by *Akt1*^+/+^;*Akt2*^+/+ ^mothers (Figure 1a).

In addition to the dramatic growth defect observed in pups nursed by *Akt1*^-/-^;*Akt2*^+/- ^mothers, these pups also displayed markedly increased perinatal mortality. By postpartum day 9, 70% of pups nursed by *Akt1*^-/-^;*Akt2*^+/- ^mice had died, compared with 30% of pups nursed by *Akt1*^-/-^;*Akt2*^+/+ ^mice and 2% of pups nursed by wild-type mice (Figure 1b).

Consistent with the severe growth retardation and increased mortality of pups nursed by *Akt1*^-/-^;*Akt2*^+/- ^mice, milk production was significantly decreased in these mice (Figure 1c). Compared with wild-type mice, *Akt1*^-/-^;*Akt2*^+/- ^mice displayed a nine-fold reduction in oxytocin-stimulated milk secretion (*P *= 0.0001) (Figure 1c), a reduction even more profound than the four-fold reduction observed in *Akt1*^-/-^;*Akt2*^+/+ ^mice (Figure 1c). These observations suggest that milk production in the lactating mammary gland is influenced by allele dosages of *Akt*.

### Akt is required for mammary differentiation during pregnancy and lactation

Milk production is the culmination of an orchestrated series of developmental events. Exposed to the hormonal milieu of pregnancy and lactation, mammary epithelial cells proliferate to form alveoli and differentiate into milk-secreting cells. *Akt1*^-/-^;*Akt2*^+/+ ^mice exhibit a lactation defect, yet mammary glands from these mice undergo normal alveologenesis and secretory differentiation during pregnancy [25]. Since *Akt1*^-/-^;*Akt2*^+/- ^mice displayed a more severe lactation defect than *Akt1*^-/-^;*Akt2*^+/+ ^mice, we examined alveolar development and differentiation in these mice.

**Figure 2:**
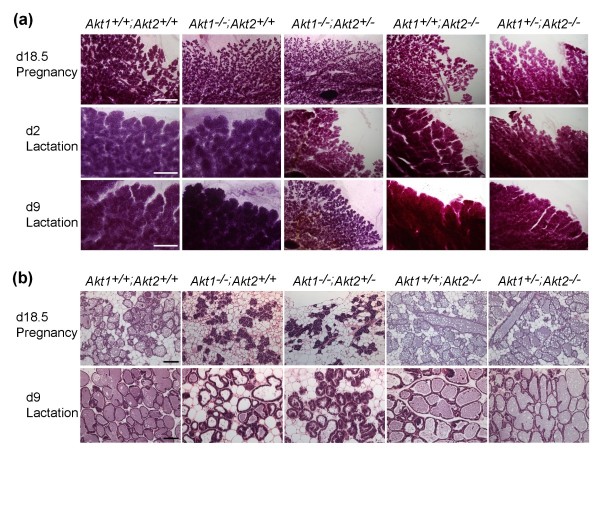
***Akt1*^-/-^;*Akt2*^+/- ^mice exhibit defective expansion of mammary gland during late pregnancy and lactation**. **(a) **Whole-mount carmine-stained mammary glands from *Akt1*^+/+^;*Akt2*^+/+ ^mice, *Akt1*^-/-^;*Akt2*^+/+ ^mice, *Akt1*^-/-^;*Akt2*^+/- ^mice, *Akt1*^+/+^;*Akt2*^-/- ^mice, and *Akt1*^+/-^;*Akt2*^-/- ^mice at day 18.5 of pregnancy and days 2 and 9 of lactation. **(b) **H & E-stained sections of mammary glands from mice with indicated *Akt *genotypes at day 18.5 of pregnancy (top) and day 9 of lactation (bottom). Scale bars: (a) 2 mm, (b) 100 μm.

Examination of carmine-stained whole mounts of mammary glands harvested from late-term pregnant and lactating mice revealed a defect in the expansion of lobuloalveolar structures in *Akt1*^-/-^;*Akt2*^+/- ^mice compared with *Akt1*^-/-^;*Akt2*^+/+ ^mice or wild-type mice (Figure 2a). By day 9 of lactation, *Akt1*^-/-^;*Akt2*^+/- ^mammary epithelia failed to form fully-expanded alveolar secretory units (Figure 2a). Analysis of histological sections revealed that alveoli of *Akt1*^-/-^;*Akt2*^+/+ ^mice and *Akt1*^-/-^;*Akt2*^+/- ^mice were markedly less distended with milk compared with those of wild-type mice from day 18.5 of pregnancy through day 9 of lactation (Figure 2b). By comparison, both *Akt1*^+/+^;*Akt2*^-/- ^and *Akt1*^+/-^;*Akt2*^-/- ^mammary epithelia displayed normal alveologenesis (Figure 2a,b). Evaluation of mammary sections for bromodeoxyuridine (BrdU) incorporation and TUNEL staining revealed normal rates of proliferation and apoptosis in *Akt1*^-/-^;*Akt2*^+/- ^glands during mid-to-late pregnancy (data not shown). This observation suggests that the defect in lobuloalveolar expansion in *Akt1*^-/-^;*Akt2*^+/- ^mice is due to defective functional differentiation of mammary epithelial cells, rather than reduced proliferation or survival.

**Figure 3:**
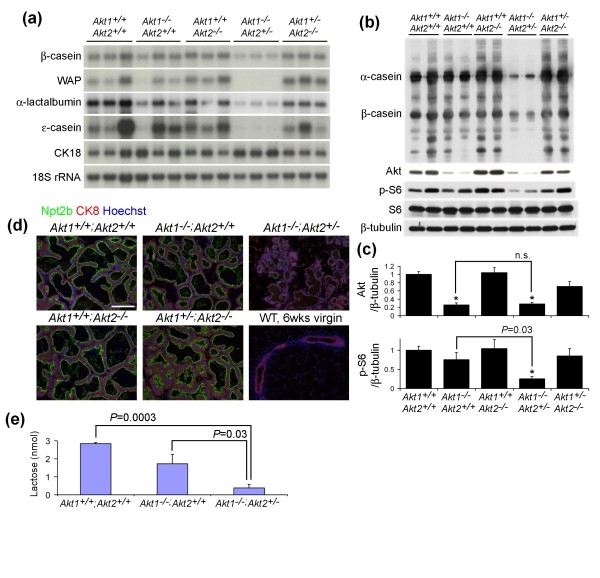
***Akt1*^-/-^;*Akt2*^+/- ^mice exhibit impaired secretory epithelial differentiation during late pregnancy and lactation**. **(a) **Northern analysis of milk protein gene expression in mammary glands from pregnant mice (day 18.5) with the indicated *Akt *genotypes. Cytokeratin 18 (CK18) and 18S rRNA were included as controls for epithelium-specific expression and RNA loading, respectively. WAP, whey acidic protein. **(b) **Representative western analysis of mammary glands from mice with the indicated *Akt *genotypes at day 18.5 of pregnancy. Protein lysates were immunoblotted with the indicated antibodies. β-Tubulin levels served as a loading control. **(c) **Quantitative analysis of Akt/β-tubulin and phospho-S6 (p-S6)/S6 in late-pregnant mice (*n *= 6 mice per genotype). The ratios were normalized to *Akt1*^+/+^;*Akt2*^+/+ ^mice. Statistical analysis in differential expression was calculated by comparing each group with *Akt1*^+/+^;*Akt2*^+/+ ^mice, except for the indicated *P *values shown between *Akt1*^-/-^;*Akt2*^+/+ ^mice and *Akt1*^-/-^;*Akt2*^+/- ^mice. *P < 0.001. n.s., not significant. **(d) **Immunofluorescence analysis of Na-Pi cotransporter 2B (Npt2b) expression during lactation. Mammary sections from lactating mice with indicated *Akt *genotypes were immunostained for Npt2b (green). Nuclei were counterstained with Hoechst 33258 (blue). Luminal epithelial cells were counterstained with CK8 (red). Mammary gland tissue from a 6-week-old virgin MTB mouse served as a negative control for staining. Scale bar: 100 μm. **(e) **Lactose levels of mammary tissues from *Akt1*^+/+^;*Akt2*^+/+ ^mice, *Akt1*^-/-^;*Akt2*^+/+ ^mice, and *Akt1*^-/-^;*Akt2*^+/- ^mice at day 9 of lactation (*n *= 4 for each genotype).

The morphological and histological signs of reduced milk secretion in *Akt1*^-/-^;*Akt2*^+/- ^mammary glands suggested that the alveolar epithelia failed to undergo functional differentiation. To determine the differentiation status of *Akt1*^-/-^;*Akt2*^+/- ^mammary epithelia we examined the expression of milk protein genes. Sequential upregulation of early (*β-casein*), mid (*WAP *and *α-lactalbumin*), and late (*ε-casein*) milk protein genes is a hallmark of secretory differentiation of the mammary epithelium [27]. Northern analysis of mammary gland mRNA at day 18.5 of pregnancy demonstrated that expression of *β-casein*, *WAP*, and *ε-casein *was not altered in *Akt1*^-/-^;*Akt2*^+/+ ^mice or in *Akt1*^+/+^;*Akt2*^-/- ^mice. Expression of these genes was significantly reduced, however, in *Akt1*^-/-^;*Akt2*^+/- ^mice (Figure 3a). Consistent with these data, immunoblotting analysis revealed markedly reduced levels of milk proteins in the mammary gland of *Akt1*^-/-^;*Akt2*^+/- ^mice at day 18.5 of pregnancy (Figure 3b).

The failure of mammary glands from *Akt1*^-/-^;*Akt2*^+/- ^mice to express milk proteins suggested a defect in secretory differentiation. Npt2b, a Na-Pi co-transporter, is a marker of secretory differentiation and is highly expressed in the lactating, but not nulliparous, mammary gland [17,25,28,29]. We therefore examined the expression of Npt2b in mammary tissue from lactating mice of differing *Akt *genotypes. Whereas Npt2b expression was appropriately upregulated in lactating mammary epithelia of wild-type mice, *Akt1*^-/-^;*Akt2*^+/+ ^mice, *Akt1*^+/+^;*Akt2*^-/- ^mice, and *Akt1*^+/-^;*Akt2*^-/- ^mice, Npt2b expression failed to be upregulated in lactating *Akt1*^-/-^;*Akt2*^+/- ^mice (Figure 3d). Notably, total Akt protein expression was significantly lower in the mammary glands of *Akt1*^-/-^;*Akt2*^+/+ ^mice compared with *Akt1*^+/+^;*Akt2*^-/- ^mice (0.26 ± 0.05 vs. 1.04 ± 0.13; Figure 3b,c), indicating that Akt1 is the predominant form of Akt in the mammary gland at day 18.5 of pregnancy. Consistent with this notion, total Akt expression was significantly reduced by deletion of one allele of *Akt1 *in *Akt2*^-/- ^mice (0.71 ± 0.12 for *Akt1*^+/-^;*Akt2*^-/- ^mice vs. 1.04 ± 0.13 for *Akt1*^+/+^;*Akt2*^-/- ^mice), but not by deletion of one allele of *Akt2 *in *Akt1*^-/- ^mice (0.29 ± 0.04 for *Akt1*^-/-^;*Akt2*^+/- ^mice vs. 0.26 ± 0.05 for *Akt1*^-/-^;*Akt2*^+/+ ^mice) (Figure 3b,c). Taken together, these results indicate that the severe lactation defect observed in *Akt1*^-/-^;*Akt2*^+/- ^mice is caused by defective secretory differentiation of the mammary gland due to a complete lack of Akt1 in conjunction with partial loss of Akt2.

### Akt regulates the production of essential milk components in the lactating mammary gland

Milk is a complex mixture of proteins, lipids and carbohydrates [30]. Our finding that milk protein expression is reduced in *Akt1*^-/-^;*Akt2*^+/- ^mice (Figure 3a,b), together with our previous finding that Akt1 is required for lipid synthesis during lactation [25], led us to examine potential roles for Akt in the biosynthesis of each of the three components of milk.

The cellular capacity for protein synthesis is regulated by the mammalian target of rapamycin (mTOR) pathway. The decrease in milk proteins observed in *Akt1*^-/-^;*Akt2*^+/- ^mammary glands could be due to the observed decreases in steady-state levels of transcription for milk protein genes (Figure 3a), or due to decreased mRNA expression coupled with decreased rates of translation for milk proteins. To address this issue, we examined levels of phospho-S6, a direct downstream target of the mTOR pathway, in the late pregnant mammary gland. Levels of phospho-S6 were significantly reduced in the mammary glands of *Akt1*^-/-^;*Akt2*^+/- ^mice compared with either wild-type mice (*P *< 0.001) or *Akt1*^-/-^;*Akt2*^+/+ ^mice (*P *= 0.03), indicating that mTOR activity is decreased in the *Akt1*^-/-^;*Akt2*^+/- ^mammary gland (Figure 3b,c).

Lactose in mammary alveolar cells is synthesized from glucose and galactose by the lactose synthase complex, which consists of α-lactalbumin and galactosyltransferase [31]. In *Akt1*^-/-^;*Akt2*^+/+ ^mammary tissue, a modest decrease in intraepithelial lactose levels was evident (Figure 3e), presumably due to reduced glucose uptake coupled with normal levels of *α-lactalbumin *(Figure 3a) [25]. In contrast, lactose levels were dramatically reduced in *Akt1*^-/-^;*Akt2*^+/- ^mice during lactation, reflecting decreased glucose uptake coupled with a marked decrease in *α-lactalbumin *expression (*P *= 0.0003 and *P *= 0.03 compared with wild-type mice and *Akt1*^-/-^;*Akt2*^+/+ ^mice, respectively) (Figure 3a,e).

**Figure 4:**
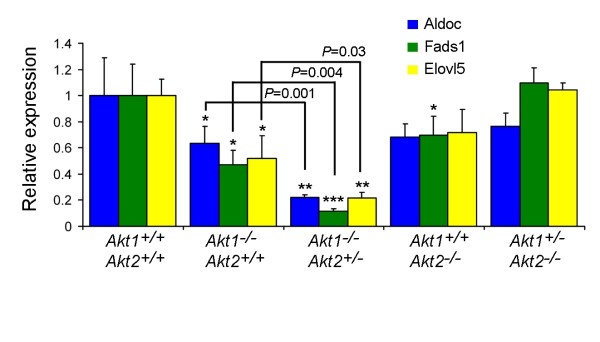
**Expression of lipid synthetic enzymes is markedly decreased in *Akt1*^-/-^;*Akt2*^+/- ^mice**. Relative expression of *Aldoc*, *Fads1 *and *Elovl5 *mRNA in mammary glands from Akt knockout mice at day 9 of lactation (*n *= 4 for each genotype). Average expression values normalized to *cytokeratin 18 *± standard error of the mean are shown. Statistical differences in expression were calculated by comparing each group with *Akt1*^+/+^;*Akt2*^+/+ ^mice except the indicated *P *values shown between *Akt1*^-/-^;*Akt2*^+/+ ^mice and *Akt1*^-/-^;*Akt2*^+/- ^mice. *P < 0.05. **P < 0.01. ***P < 0.001.

We previously showed that *Akt1 *contributes to lipid biosynthesis in the lactating mammary gland by regulating expression of genes involved in lipid metabolism, such as *Scd2*, *Scd3 *and *Dgat2 *[25]. To extend this analysis, we examined expression of *Aldoc*, *Fads1 *and *Elovl5*, which are preferentially expressed in the mammary epithelium, are upregulated during lactation and have been implicated in fatty acid synthesis [32]. Quantitative RT-PCR analysis demonstrated that the expression levels of *Aldoc*, *Fads1 *and *Elovl5 *were lower in *Akt1*^-/-^;*Akt2*^+/+ ^mice compared with wild-type mice, and were further reduced in *Akt1*^-/-^;*Akt2*^+/- ^mice (Figure 4). In aggregate, these findings demonstrate that Akt is required for production of the three main components of milk - milk proteins, lipid, and lactose - in the lactating mammary gland.

### Akt is required for Stat5 activation

The Prlr-Jak2-Stat5 signaling pathway plays a critical role in alveolar morphogenesis and differentiation [11,16,18,33,34], as illustrated by the fact that *Stat5*-deficient mice fail to form alveoli during pregnancy and do not express milk protein genes [17]. Although alveolar formation in *Akt1*^-/-^;*Akt2*^+/- ^mice occurred normally, the defect in mammary epithelial differentiation observed in *Akt1*^-/-^;*Akt2*^+/- ^mice led us to hypothesize that a functional relationship might exist between the Akt and Stat5 pathways.

**Figure 5:**
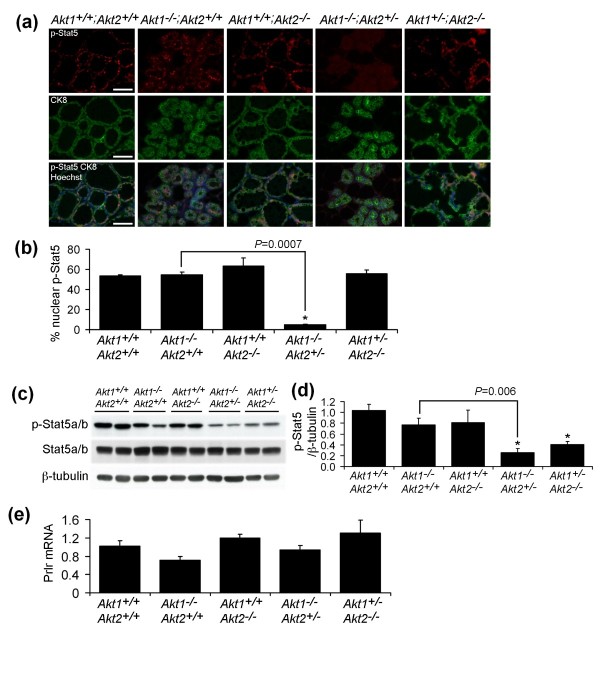
**Stat5a/b activation is reduced in the mammary glands of *Akt1*^-/-^;*Akt2*^+/- ^mice**. **(a) **Immunofluoresence analysis of phospho-Stat5a/b (p-Stat5) expression in mammary tissues from day 18.5 pregnant mice with the indicated *Akt *genotypes. Luminal epithelial cells and nuclei were counterstained with anti-CK8 (green) and Hoechst 33258 (blue), respectively. Scale bars represent 50 μm. **(b) **Quantitative analysis of nuclear p-Stat5 in mammary epithelial cells of late pregnant mice (*n *= 3 mice per genotype). Statistical analysis in differential activity was calculated by comparing each group with *Akt1*^+/+^;*Akt2*^+/+ ^mice, except for the indicated *P *value shown between *Akt1*^-/-^;*Akt2*^+/+ ^mice and *Akt1*^-/-^;*Akt2*^+/- ^mice. *P < 0.0001. **(c) **Representative western analysis of p-Stat5a/b and total Stat5a/b expression in mammary tissues from day 18.5 pregnant mice with indicated *Akt *genotypes. β-Tubulin levels served as a loading control. **(d) **Quantitative analysis of p-Stat5/Stat5 in late-pregnant mice (*n *= 6 mice per genotype). The ratio was normalized to *Akt1*^+/+^;*Akt2*^+/+ ^mice. Statistical analysis in differential activity was calculated by comparing each group with *Akt1*^+/+^;*Akt2*^+/+ ^mice, except for the indicated *P *values shown between *Akt1*^-/-^;*Akt2*^+/+ ^mice and *Akt1*^-/-^;*Akt2*^+/- ^mice. *P < 0.001. **(e) **Relative expression of *Prlr *mRNA in mammary glands from *Akt *knockout mice at day 18.5 of pregnancy (*n *= 4 for each genotype). Average expression values normalized to *cytokeratin 18 *± standard error of the mean are shown.

To address this hypothesis, we evaluated Stat5 activity in the mammary glands of *Akt1*^-/-^;*Akt2*^+/- ^pregnant mice by determining the fraction of epithelial cells with nuclear phospho-Stat5a/b. Mammary tissue was harvested from mice with various *Akt *genotypes at day 18.5 of pregnancy and immunofluorescence was performed for phospho-Stat5a/b and cytokeratin 8. This revealed that the fraction of mammary epithelial cells with nuclear phospho-Stat5a/b was markedly diminished during pregnancy in *Akt1*^-/-^;*Akt2*^+/- ^mice compared with *Akt1*^-/-^;*Akt2*^+/+ ^mice, *Akt1*^+/+^;*Akt2*^-/- ^mice, *Akt1*^+/-^;*Akt2*^-/- ^mice, or wild-type mice (Figure 5a,b). The percentage of mammary epithelial cells with nuclear phospho-Stat5a/b was 10-fold lower in *Akt1*^-/-^;*Akt2*^+/- ^mice compared with wild-type mice or *Akt1*^-/-^;*Akt2*^+/+ ^mice (*P *< 0.0001 and *P *= 0.0007, respectively). Consistent with this, immunoblotting revealed markedly decreased levels of phospho-Stat5a/b in the mammary glands of pregnant *Akt1*^-/-^;*Akt2*^+/- ^mice (Figure 5c,d). In contrast, phospho-Stat5a/b levels were unaffected by deletion of *Akt1 *or *Akt2 *alone (Figure 5c,d).

Of note, phospho-Stat5 levels were decreased in *Akt1*^+/-^;*Akt2*^-/- ^mice, although not to the extent observed in *Akt1*^-/-^;*Akt2*^+/- ^mice as normalized phospho-Stat5 levels in *Akt1*^+/-^;*Akt2*^-/- ^mice remained ~60% higher than in their *Akt1*^-/-^;*Akt2*^+/- ^counterparts (Figure 5d). Interestingly, despite the modest decreases in phospho-Stat5 levels observed in *Akt1*^+/-^;*Akt2*^-/- ^mice by western blotting, the fraction of alveolar epithelial cells exhibiting nuclear phospho-Stat5 observed by immunofluorescence was unaffected in these mice (Figure 5b,d). Consistent with the nuclear localization of phospho-Stat5 representing the most reliable indicator of its functional status as a transcription factor, milk production, milk protein gene expression, mTOR activity, Npt2b upregulation, and expression of lipid synthetic enzymes were all normal in *Akt1*^+/-^;*Akt2*^-/- ^mice. As such, the normal nuclear localization and function of phospho-Stat5 in *Akt1*^+/-^;*Akt2*^-/- ^mice despite modest reductions in total phospho-Stat5 levels could reflect a threshold requirement for Akt for phospho-Stat5 nuclear localization, effects of Akt deletion on phospho-Stat5 levels in the adipose stroma, or the intriguing possibility that Akt may affect the nuclear localization of Stat5 by mechanisms other than its activation by phosphorylation.

Quantitative RT-PCR analysis revealed that that *Prlr *expression in *Akt1*^-/-^;*Akt2*^+/- ^mice was comparable with that observed in wild-type mice (Figure 5e). This indicates that reduction in Stat5 activity in *Akt1*^-/-^;*Akt2*^+/- ^mice is not due to a defect in prolactin receptor expression. Together, these findings demonstrate that Akt is required for Stat5a/b activation in the mammary gland during pregnancy.

### Akt regulates the expression of regulators of prolactin-Jak-Stat5 signaling

A number of molecules have been identified that regulate activity of the Prlr-Jak-Stat5 pathway. For example, caveolin-1 and Socs2 function as suppressors of prolactin-induced phosphorylation of Stat5a/b in mammary epithelial cells, whereas Id2 positively regulates Stat5a/b signaling [35-39]. Id2 is also essential for mammary gland development during pregnancy and lactation [37,38]. In addition, recent experiments have demonstrated that Elf5 expression in the mammary epithelium can rescue the alveolar defect observed in *Prlr*^-/- ^mice and can drive pregnancy-associated mammary differentiation [35,40].

**Figure 6:**
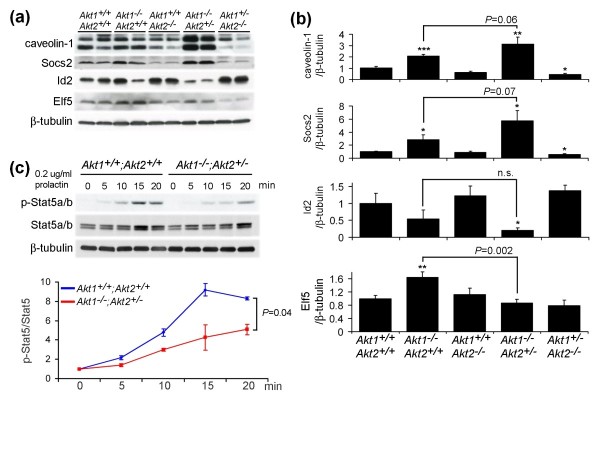
**Akt regulates the expression of key regulators of Stat5 activity**. **(a) **Representative western analysis of expression of proteins that regulate mammary differentiation. Mammary protein lysates from mice with indicated *Akt *genotypes at day 18.5 of pregnancy. β-Tubulin levels served as a loading control. **(b) **Quantitative analysis of individual molecule expression in late-pregnant mice (*n *= 6 mice per genotype). The ratios in each group were normalized to *Akt1*^+/+^;*Akt2*^+/+ ^mice. Statistical analysis was calculated by comparing each group with *Akt1*^+/+^;*Akt2*^+/+ ^mice, except for the indicated *P *values shown between *Akt1*^-/-^;*Akt2*^+/+ ^mice and *Akt1*^-/-^;*Akt2*^+/- ^mice. *P < 0.05. **P < 0.01. ***P < 0.001. n.s., not significant. **(c) **Representative western analysis of Stat5a/b activation in *Akt1*^+/+^;*Akt2*^+/+ ^and *Akt1*^-/-^;*Akt2*^+/- ^mammary tissues at day 18.5 of pregnancy treated with prolactin (0.2 μg/ml) for the indicated periods (top panel). Graph shows the ratios of phospho-Stat5 (p-Stat5)/Stat5 at the indicated timepoints calculated from three independent experiments (bottom panel).

To elucidate the basis of the requirement for Akt in Prlr-Jak-Stat5 signaling, we examined levels of caveolin-1, Socs2, and Id2 in day 18.5 pregnant glands from mice bearing targeted deletions in *Akt1 *and *Akt2*. This analysis revealed that expression of the negative regulators of Stat5 signaling, caveolin-1 and Socs2, were markedly increased in *Akt1*^-/-^;*Akt2*^+/- ^mice, whereas expression of the positive regulator of Stat5 signaling, Id2, was markedly decreased compared with genetic controls (Figure 6a,b). In contrast, Elf5 expression was unaffected in *Akt1*^-/-^;*Akt2*^+/- ^mice, despite their severe lactation defect (Figure 6a,b). In addition, although animal-to-animal variation was evident (Figure 6a), caveolin-1, Socs2 and Id2 levels did not differ between wild-type mice and *Akt2*-deficient mice (Figure 6b).

Consistent with a causal relationship between changes in the expression of Stat5 regulatory molecules and changes in phospho-Stat5 levels, expression of caveolin-1 and Socs2 were highest - and expression of Id2 was lowest - in *Akt1*^-/-^;*Akt2*^+/- ^mice compared with other genotypes (Figure 6a). Changes in caveolin-1, Socs2 and Id2 expression were not observed in *Akt1*^+/-^;*Akt2*^-/- ^mice, however, despite modest reductions in phospho-Stat5 levels. This suggests the possibility that additional regulatory molecules downstream of Akt may play a role in modulating Prlr-Jak2-Stat5 signaling.

To address the hypothesis that Akt-mediated decreases in caveolin-1 and Socs2 expression, along with increases in Id2 expression, are required for prolactin-induced Stat5a/b activation and mammary differentiation during pregnancy, we evaluated the extent of prolactin-induced Stat5a/b activation *in vitro *in mammary glands harvested from *Akt1*^+/+^;*Akt2*^+/+ ^mice and *Akt1*^-/-^;*Akt2*^+/- ^mice at day 18.5 of pregnancy. Intact mammary tissues were used instead of isolated mammary epithelial cells since dissociation of the mammary gland results in loss of caveolin-1 expression (unpublished observations), despite the fact that caveolin-1 is highly expressed in both the mammary epithelium and adipocytes of the virgin mammary gland [39]. This observation suggests that the architecture of the mammary gland is important in maintaining caveolin-1 expression, which is in turn consistent with our observation that mammary epithelial cell lines express only low levels of caveolin-1 compared with the mammary gland (unpublished observations).

Addition of prolactin to organ cultures containing mammary tissue from *Akt1*^-/-^;*Akt2*^+/- ^mice or *Akt1*^+/+^;*Akt2*^+/+ ^mice revealed that peak levels of Stat5a/b activation in *Akt1*^-/-^;*Akt2*^+/- ^glands were less than one-half of those observed in wild-type mice (Figure 6c). These results indicate that prolactin-induced Stat5a/b activation in the pregnant mammary gland is markedly blunted in *Akt1*^-/-^;*Akt2*^+/- ^mice. In aggregate, these findings suggest that Akt potentiates prolactin-induced Stat5a/b activation and mammary epithelial differentiation, at least in part by downregulating the known suppressors of Stat5a/b phosphorylation, caveolin-1 and Socs2, and by upregulating the positive regulator of Stat5a/b signaling, Id2.

## Discussion

The prolactin-Jak-Stat5 pathway has long been recognized as a central mediator of pregnancy-induced lobuloalveolar development and lactation, which together constitute a developmental transition that is essential for the survival of mammals. Accordingly, the role of this pathway in mammary development has been intensively studied. In the present manuscript we describe a previously unrecognized requirement for Akt in Prlr-Jak-Stat5 signaling. Mice lacking one allele of *Akt2 *and both alleles of *Akt1 *displayed a severe lactation defect due to the global impairment of alveolar epithelial cell differentiation. Consistent with their failure to terminally differentiate, pregnant *Akt1*^-/-^;*Akt2*^+/- ^mice fail to upregulate Npt2b or phospho-Stat5a/b and display markedly reduced synthesis of each of the three major components of milk during lactation. Notably, epithelial cell proliferation, cell survival and the formation of architecturally normal acini during pregnancy were unaffected by Akt deletion, reinforcing that the lactation defect observed in *Akt*^-/-^;*Akt2*^+/- ^mice results from a defect in differentiation, rather than from a failure to form acinar structures. In aggregate, these findings establish an essential but heretofore unrecognized role for Akt in epithelial differentiation.

Despite the fact that both *Akt1*^-/-^;*Akt2*^+/+ ^mice and *Akt1*^-/-^;*Akt2*^+/- ^mice exhibit defects in lactation, the molecular basis of their lactation phenotypes is strikingly different. The isoform-specific defect in lactation observed in *Akt1*-deficient mice occurs in the absence of defects in differentiation and results from a defect in metabolism [25]. This metabolic defect is due to the failure of *Akt1*-deficient mammary epithelial cells to upregulate key Akt1 target genes, most notably the glucose transporter Glut1 and three lipid synthetic enzymes *Acly*, *Scd2*, and *Scd3*. These molecular defects result in a profound inability of terminally differentiated mammary epithelial cells to take up glucose or to synthesize normal amounts of lipid. Nevertheless, mammary epithelial differentiation is normal in lactating *Akt1*^-/- ^mice, as demonstrated by physiologically normal levels of Stat5 activation, normal upregulation of the terminal differentiation marker Npt2b, normal downregulation of the virgin-specific transporter NKCC1, normal expression of all major milk proteins, and normal intraepithelial lactose levels.

In contrast, our current study demonstrates that deletion of one allele of *Akt2 *in *Akt1*-deficient mice results in a severe defect in mammary epithelial differentiation that is due to a failure to activate Stat5. In contrast to *Akt1*-deficient mice, late-pregnant *Akt1*^-/-^;*Akt2*^+/- ^mice exhibit dramatically reduced Prlr-Jak-Stat5 signaling, as well as markedly reduced milk protein expression, lactose levels, lipid synthesis, expression of the terminal differentiation marker Npt2b, and mTOR activity.

The lactation defect observed in *Akt1*^-/- ^mice is thus due to a metabolic defect that results from a failure to upregulate Glut1 and other Akt1-specific target genes. This defect occurs in the context of normal Prlr-Jak-Stat5 signaling and normal mammary epithelial differentiation. In contrast, the lactation defect in *Akt1*^-/-^;*Akt2*^+/- ^mice is due to a profound defect in mammary epithelial differentiation that results from a failure to activate Stat5. While the outward phenotypes (that is, lactation defect) of *Akt1*^-/- ^mice and *Akt1*^-/-^;*Akt2*^+/- ^mice are similar at a superficial level, the molecular phenotypes as well as the molecular basis for these phenotypes are profoundly different.

The defect in Stat5 activation observed in *Akt1*^-/-^;*Akt2*^+/- ^mice is due, at least in part, to a failure to upregulate the positive regulator of Prlr-Jak-Stat5 signaling, Id2, or to downregulate the negative regulators of prolactin-Jak2-Stat5 signaling, caveolin-1 and Socs2. In addition, our findings suggest that Akt most likely regulates the expression or activity of other molecules that modulate Prlr-Jak-Stat5 pathway activity. Together, these findings provide a molecular basis for this previously unrecognized connection between the Akt and Stat5 pathways.

Notably, mammary epithelial proliferation and apoptosis rates were unaffected in *Akt1*^-/-^;*Akt2*^+/- ^mice during pregnancy, suggesting that Akt is essential for Stat5-dependent secretory differentiation of mammary epithelium, but possibly not for Stat5-dependent alveolar development or acinar formation; that is, whereas *Stat5*-deficiency in the mammary gland results in a failure of lobuloalveolar development as well as secretory differentiation, *Akt1*^-/-^;*Akt2*^+/- ^mice exhibit only a defect in secretory differentiation. Nevertheless, it is possible that deletion of all four *Akt1 *and *Akt2 *alleles would result in a defect in prolactin-Stat5-mediated epithelial proliferation and, thereby, lobuloalveolar development similar to that observed in *Stat5*-deficient mice. Given the perinatal lethality of combined germline deletion of *Akt1 *and *Akt2*, however, mammary-specific deletion of these genes may be required to determine the role of the remaining *Akt2 *allele in mammary epithelial proliferation and differentiation.

Elf5 has been shown recently to regulate alveolar cell differentiation by acting downstream of the prolactin receptor [41]. Since Elf5 expression was unchanged in the mammary glands of *Akt1*^-/-^;*Akt2*^+/- ^mice despite their dramatic reduction in Stat5 activity, our data suggest that the impact of Akt deletion on Prlr-Jak-Stat signaling is not mediated by Elf5. Rather, our findings suggest that Akt and Elf5 may act via parallel pathways, that Elf5 alone is not sufficient to compensate for loss of Prlr signaling, and that factors other than Prlr signaling may regulate Elf5 [19,41].

Maroulakou and colleagues have reported that *Akt1 *deficiency delayed, whereas *Akt2 *deficiency facilitated, mammary epithelial differentiation during pregnancy and lactation [42]. In contrast, consistent with our prior study, the findings described here confirm that deletion of either *Akt1 *or *Akt2 *alone has no appreciable effect on secretory differentiation during pregnancy or lactation. Moreover, our finding that deletion of one allele of *Akt2 *in *Akt1*-deficient mice results in a pronounced defect in mammary epithelial differentiation that is not observed in mice deficient for Akt1 alone strongly suggests that Akt2 synergizes with - rather than antagonizes - the pro-differentiation effects of Akt1 on mammary epithelial cells. Whether this discrepancy is explained by the mixed genetic background of mice used in the former study or other factors remains to be determined.

## Conclusions

As we have previously described, Akt1 is required for the orchestrated metabolic response of the mammary gland to lactation. We now report that the combined action of Akt1 and Akt2 is required for mammary epithelial differentiation during pregnancy and lactation due to their requirement for Stat5 activation. Together, our findings establish Akt as an essential and central regulator of epithelial differentiation and metabolism during lactation. As Akt is a common downstream effector of many receptor tyrosine kinases, the finding that the Akt and Stat5 signaling pathways are functionally interconnected has important implications for the numerous biological processes regulated by these pathways. In particular, both pathways regulate cell survival and cell proliferation, and have been implicated in mammary tumorigenesis in rodents and in humans [43-45]. Consequently, our findings predict that therapeutic blockade of the Akt pathway may be effective in treating Prlr-Jak-Stat5-driven tumors, whereas blockade of Prlr-Jak-Stat5 signaling may be effective in treating Akt-driven tumors. In light of the fact that normal pathways of differentiation and development are frequently usurped during the process of carcinogenesis, we predict that combined therapeutic approaches targeting crosstalk between the Akt and Prlr-Jak-Stat5 pathways may be particularly effective in the case of breast cancer.

## Abbreviations

Acly: ATP citrate lyase; Aldoc: aldolase C; BrdU: bromodeoxyuridine; BSA: bovine serum albumin; Dgat2: diacylglycerol *O*-acyltransferase 2; Elf5: E74-like factor 5; Elovl5: elongation of very-long-chain fatty acids protein 5; Fads1: fatty acid desaturase 1; Gata3: GATA binding protein 3; H & E: hematoxylin and eosin; Id2: inhibitor of DNA binding 2; Jak2: Janus kinase 2; mTOR: mammalian target of rapamycin; NKCC1: sodium/potassium/chloride cotransporter isoform 1; Npt2b: Na-Pi cotransporter 2B; PBS: phosphate-buffered saline; Prlr: prolactin receptor; qRT-PCR: quantitative real-time polymerase chain reaction; Scd: stearoyl-coenzyme A desaturase; SEM: standard error of the mean; Socs2: suppressor of cytokine signaling 2; Stat5: signal transducer and activator of transcription 5; TUNEL: terminal deoxynucleotidyl transferase dUTP nick end labeling; WAP: whey acidic protein.

## Competing interests

The authors declare that they have no competing interests.

## Authors' contributions

CCC participated in the design, execution and analysis of experiments and participated in drafting the manuscript. RBB participated in the execution and analysis of mouse experiments. DBS participated in the execution and analysis of immunofluorescence experiments. CPP participated in the execution of mouse experiments. RHH participated in the execution of mouse and molecular experiments. JVA participated in the analysis of data and drafting of the manuscript. MJB participated in the mouse experiments and drafting of the manuscript. LAC participated in the design and analysis of experiments and drafting of the manuscript.
